# Self-amputation of the Upper Extremity: A Case Report and Review of the Literature

**DOI:** 10.7759/cureus.5858

**Published:** 2019-10-07

**Authors:** Erik Marques, Eric J Maiorino, Zachary Tallackson, Hossein Masoomi

**Affiliations:** 1 Plastic and Reconstructive Surgery, University of Texas Health Science Center, Houston, USA

**Keywords:** self-amputation, acute psychosis, replantations

## Abstract

Indications for upper-extremity replantation include wrist-level and wrist-proximal amputations, due to the devastating loss of function incurred from these severe injuries. Decisions regarding replantation must be made expeditiously at these proximal levels in an effort to minimize ischemia time. This decision-making process becomes more complicated when a patient presents following intentional self-amputation of an extremity, especially in the setting of an associated mood disorder, psychiatric illness, and/or frank psychosis. A case report is presented involving a 28-year-old right-hand dominant male with untreated depression and recent suicidal ideation who sustained a complete left forearm amputation (distal-third forearm-level) from a self-inflicted circular saw injury. We conducted a PubMed literature search of other reported cases of intentional self-amputations of the hand and upper extremity. The patient underwent replantation of the left upper extremity. At six years postoperatively, the patient was extremely satisfied with the appearance and function of the replanted extremity. Dash score was 5.8 with a Chen Grade 1 (excellent) functional recovery. A literature search identified 16 cases of self-inflicted upper extremity amputation. One patient died at the scene. 87% (13/15) of patients presenting to the hospital were diagnosed with a psychiatric disorder (depression n = 6, bipolar n = 2, and schizophrenia n = 5). 67% (10/15) of these patients were also diagnosed with psychosis. Ten patients underwent replantation (nine at hand/wrist level and one at forearm level), all of which were viable postoperatively. Detailed functional outcome data were not reported in any of the cases. Four patients (40%) were pleased or satisfied with the outcome, but subjective outcomes were not reported for the other six patients. Intentional self-amputation of the hand/upper extremity is an extreme and uncommon act, often presenting with complex psychiatric issues. Although replantation is technically feasible in this patient population, long-term subjective and objective functional outcomes are largely unknown. Future study of this unique group of patients is needed to better assess patient-reported outcomes and functional outcomes of replantation, which could help guide decision making at the time of initial injury.

## Introduction and background

Traumatic amputations of the upper extremity are devastating, life-changing injuries that have substantial psychological, functional, and socioeconomic sequela [1-3]. The indications for replantation are well described and have been developed based on decades of experience [4-6]. Included among the indications for replantation are wrist-level and wrist-proximal amputations, due to the devastating loss of function incurred from these severe injuries. Decisions regarding replantation must be made expeditiously, especially at these more proximal levels in an effort to minimize ischemia time and muscle degradation. In addition to considering ischemia time (warm versus cold), other factors that may influence the decision to proceed or abort upper extremity replantation include mechanism of injury (sharp versus crush/avulsion), extent of contamination, presence versus absence of multi-level injury, and the overall medical status of the patient [4-5,7].

The decision-making process becomes even more complicated when a patient presents following the intentional self-amputation of a potentially replantable extremity. This unique patient population may present with an associated mood disorder, psychiatric illness and/or frank psychosis. Included among the relative contraindications to replantation is self-amputation, associated mental instability, or possibly an inability/unwillingness to comply with the involved postoperative therapy regimen [1,4-7]. Although patients with self-inflicted upper extremity amputations may present with one or more of the aforementioned relative contraindications, and would likely have digits discarded in a similar scenario, an issue to consider is whether the decision to revise or re-implant a more proximal injury be given a separate set of criteria. In addition, current mental state and treatment options for psychosis should be considered, attempting to take into account the patient’s expected mental state if these were to be well-controlled. The responsible expenditure of healthcare resources must be considered as well, similar to all cases where replantation is entertained. With the rarity of self-inflicted major upper extremity amputations, there are few recommendations guiding the approach and management of these uncommon events.

We report a case involving a 28-year-old male with untreated depression who presented with a forearm-level complete amputation from a deliberate, self-inflicted circular saw injury. He underwent successful replantation and functional recovery of the extremity. In addition, we conducted a literature search with the intention of providing additional insight into the management of these major, self-inflicted upper extremity injuries.

Case Report

**Figure 1:**
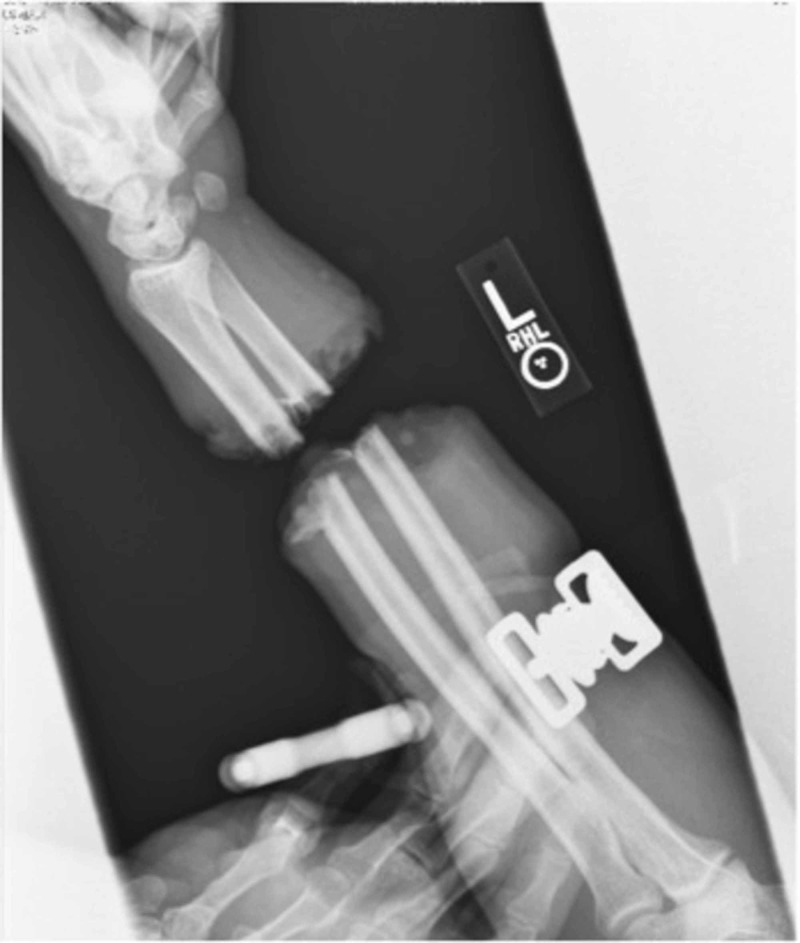
Forearm X-rays Forearm X-rays demonstrating a transverse radius and ulna forearm fracture at the mid-forearm location. X-rays demonstrate little comminution at the fracture sites.

**Figure 2:**
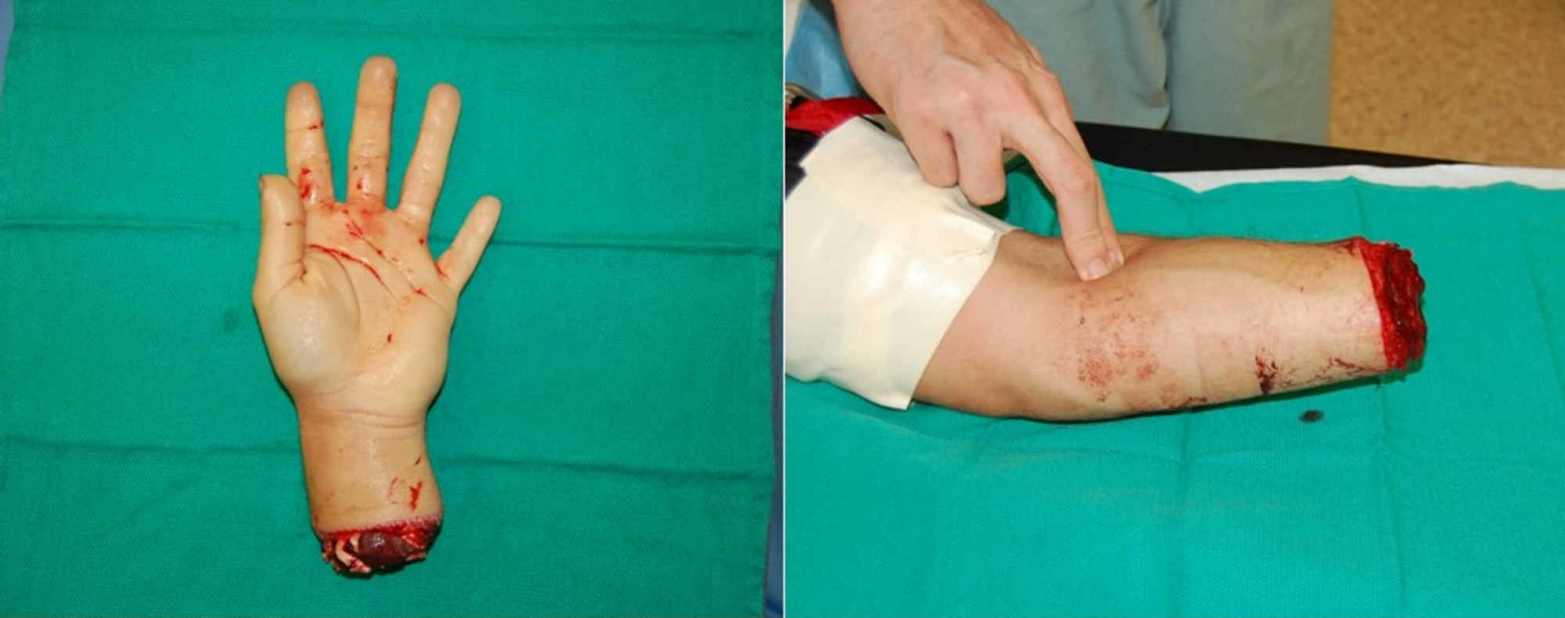
Amputated segment Image demonstrating the level of self-amputation approximately 4 cm proximal to the wrist. Sharp, clean amputation sustained from a circular saw.

A 28-year-old right-hand dominant male with a history of untreated depression and recent suicidal ideation presented with a complete amputation of the left upper extremity (forearm-level) as the result of an intentional, self-inflicted injury with a circular saw following an argument with his wife (Figure 1). The trauma occurred 45 minutes prior to arrival at our Level I trauma center with the amputated limb appropriately protected and cooled on an ice slurry. The amputation was noted to be a relatively sharp injury at the distal-third forearm-level (Figure 2). Despite a relatively flat affect, the patient appeared to recognize the gravity of the situation, seemed remorseful for his impulsive action, and desired an attempt at replantation. Emergency psychiatry consultation was not obtained because the in-house evaluation was not immediately available, and we did not want to delay operative intervention.

**Figure 3:**
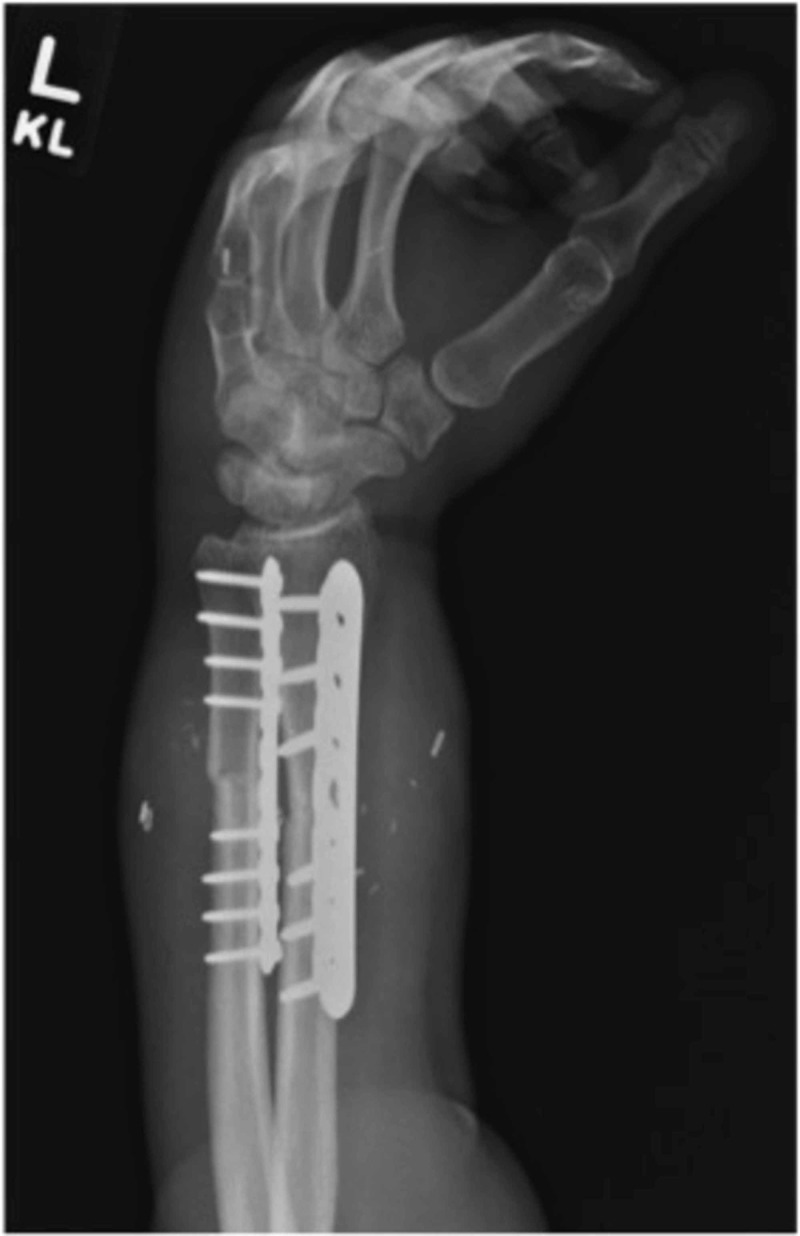
Oblique X-rays after plating Oblique X-rays demonstrating the radius and ulna forearm fixation after compression plating. Radius and ulna were both shortened by approximately 4 cm to allow for a tension-free repair of the vital soft tissues.

**Figure 4:**
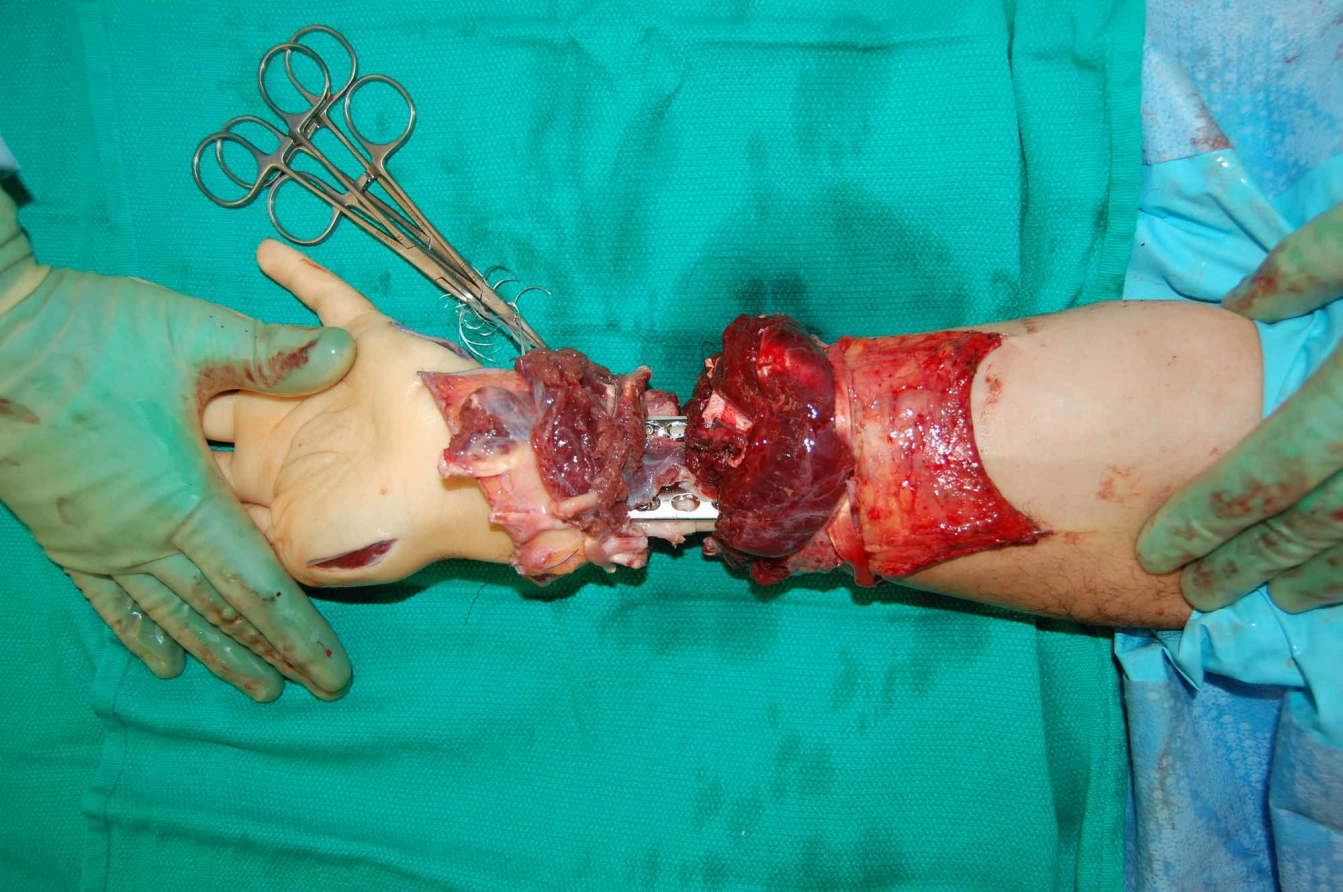
Intraoperative fixation Intraoperative image demonstrating plate and screw fixation of forearm bones

The patient underwent emergent replantation of the left upper extremity. Bony stabilization was obtained using plate and screw fixation (Figure 3). The radius and ulna were shortened by 4 cm to facilitate primary tension-free repairs of all neurovascular and tendinous structures (Figure 4). Arterial inflow was restored with ulnar artery repair and venous outflow was accomplished with anastomosis of an ulnar artery vena comitans, radial artery vena comitans, and the cephalic vein. The median nerve, ulnar nerve and superficial branch of the radial nerve were repaired primarily. All of the flexor and extensor tendons were repaired using modified Kessler four-strand core sutures. Total ischemia time was not recorded however a tourniquet was used for four hours and 28 minutes, while the arm was prepared for replantation and was let down after bony stabilization. Prophylactic hand fasciotomies were performed. Postoperatively, the patient was monitored on the microsurgery unit and psychiatry consultation was obtained. He was diagnosed with depression without psychosis. Recommendations included mood-stabilizing medication (paroxetine), one-to-one monitoring, and transfer to an inpatient psychiatric facility when cleared by the Plastic Surgery service.

On postoperative day six, the patient was returned to the operating room for closure of the hand fasciotomy wounds and split thickness skin grafting of residual forearm wounds. Hand therapy was initiated after confirmation of good skin graft take and was continued throughout the hospitalization. The patient’s depressed mood stabilized, and he demonstrated a good understanding and commitment to his hand therapy regimen while in-house. Length of hospitalization was 19 days prior to discharge to an inpatient psychiatric facility. The patient continued with hand therapy over the next several months. While all joints remained supple, he developed flexor and extensor tendon adhesions, and later underwent flexor and extensor tenolysis at six months after replantation.

**Figure 5:**
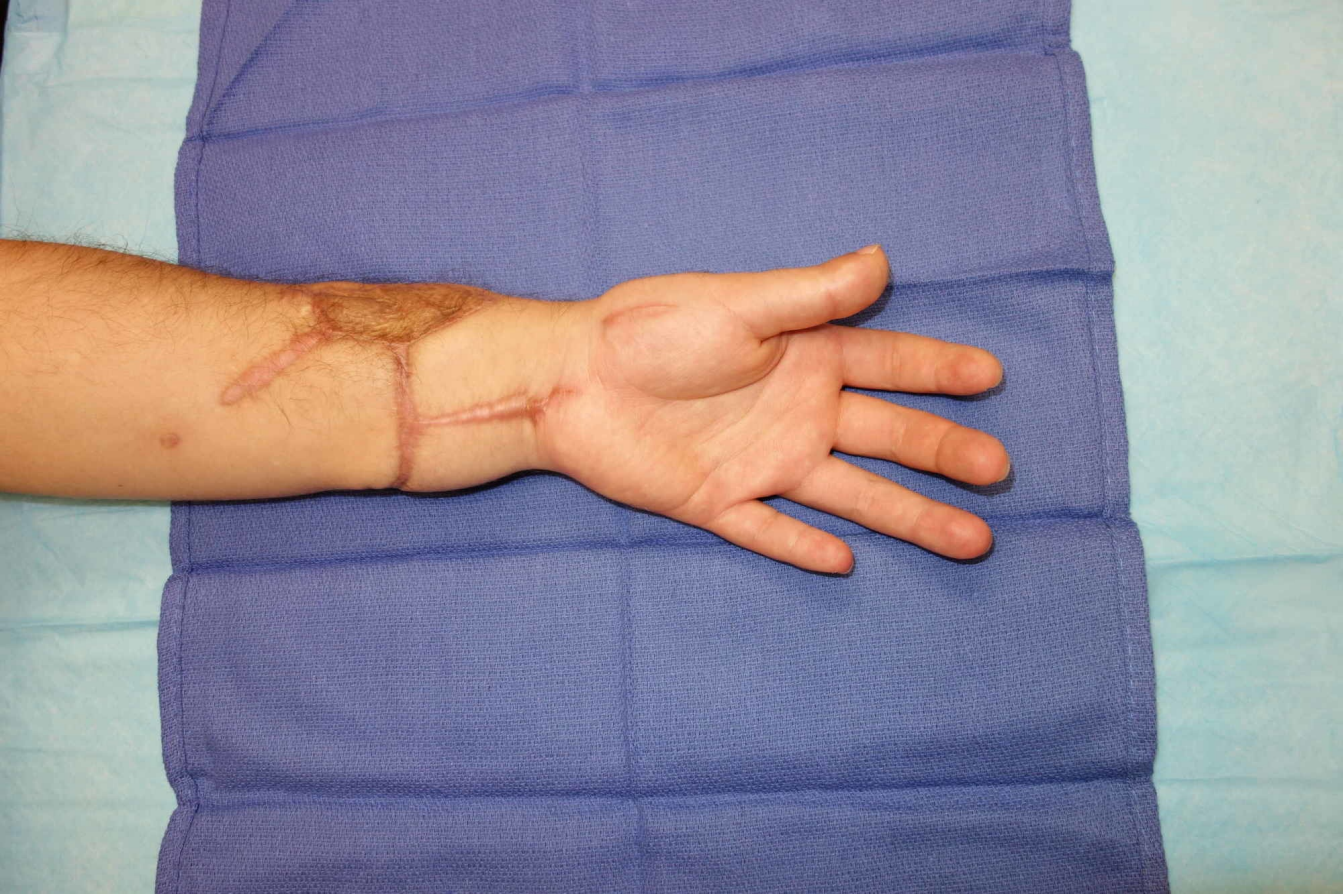
Postoperative outcome Image depicting the palmar aspect of the hand and forearm six years postoperative, demonstrating the moderate hypertrophic scarring from the patient’s previous procedures. These scars did not affect his overall functional outcome.

**Figure 6:**
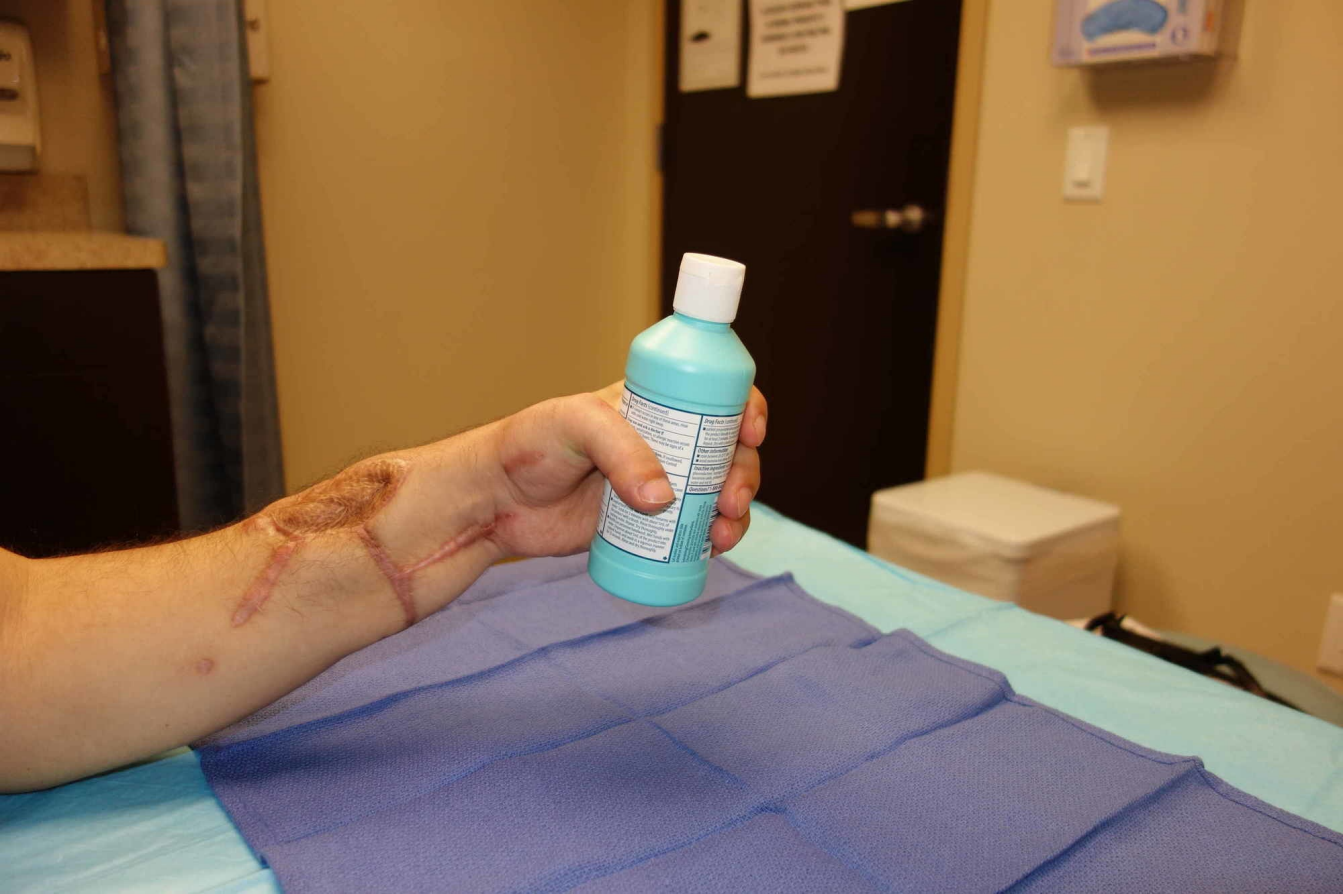
Postoperative functional outcome Patient demonstrating prehensile function of the replanted hand

**Figure 7:**
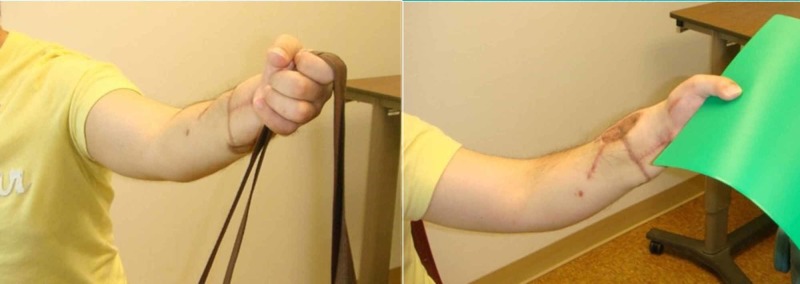
Postoperative fine motor outcome Patient demonstrating hook grasp and pinch function

At six years postoperatively, the patient was extremely satisfied with the function and appearance of the replanted extremity (despite the development of hypertrophic scarring) (Figure 5). He demonstrated good composite flexion and extension, sensory recovery of 7-9 mm two-point discrimination to all fingers and thumb and incomplete recovery of intrinsic function with functional thumb opposition (Figure 6). He has returned to his previous employment as a laboratory technician without restrictions. Dash score was 5.8 with a Chen Grade I (excellent) functional recovery (Figure 7). From a psychiatric standpoint, he was continued on paroxetine with improvement in his depressive symptoms. 

Methods

Institutional Review Board authorization was waived for this study, although individual written consent by the patient was obtained. We performed a PubMed literature search of self-inflicted hand/upper extremity amputations. We confined our search to English language publications involving wrist-level and wrist-proximal amputations. The search terms used were “self-amputation and hand” and “self-amputation and upper extremity”. Self-amputations distal to the wrist-level such as those involving the thumb or digit(s) were excluded. References of relevant papers were reviewed to capture any additional cases and reviews not obtained from the primary search terms. Cases were not included when demographics and/or clinical data were not available, such as in cases of self-amputation that may have been a part of larger series of upper limb amputations. We collected data on patient demographics (including the presence of any psychiatric co-morbidities), mechanism of injury, level of amputation, and whether replantation was performed. In the replantation subgroup, the additional recorded data included: hand/limb viability following replantation, inpatient psychiatry admission postoperatively, hand therapy compliance, follow-up time period, and any available functional outcomes or subjective patient outcomes.

## Review

Results

Based on the literature search, 16 cases of self-inflicted upper extremity amputations were identified (Table 1). Thirteen of the cases (81%) were published in the psychiatry literature with only one of the cases reported in a surgery journal [8]. The age of the patients ranged from 20-55 years with a mean of 34.2 years. Males represented 13 of the cases (81%) of self-inflicted amputation and females in three cases (19%). Mechanism of injury included saw (seven cases, 43.75 %), knife (six cases, 37.5 %), and axe (three cases, 18.75 %). Level of amputation was present at the hand/wrist level in 11 cases (68.75 %), forearm level in four cases (25%), and the upper arm in one case (6.25 %).

**Table 1:** Patient characteristics The patient characteristics of reported cases of hand & upper-extremity self-amputations available in the literature. M, male; F, female; R, right; L, left; Y, yes; N, no; N/A, not applicable; NR, not required [8-19]

Case #	Age	Sex	Side	Mechanism	Level of Amputation	Psych Consult (Y/N)	Psychiatric Diagnosis	Psychosis (Y/N)	Replant (Y/N)	References	Journal
1	23	M	R	Knife	Wrist	NR	NR	NR	Y	Jaffe (1975)	Medical
2	55	M	R	Circular saw	Wrist	Y	Psychotic Depression	Y	Y	Stewart (1980)	Psych
3	37	F	R	Power saw	Wrist	Y	Depression	NR	Y	Stewart (1980)	Psych
4	22	M	R	Axe	Hand	N	Paranoid schizophrenia	Y	Y	Hall (1981)	Psych
5	34	M	L	Axe	Distal forearm	NR	Schizophrenia	Y	Y	DeMuth (1983)	Psych
6	37	F	L	Knife	Hand	Y	Schizophrenia	Y	Y	DeMuth (1983)	Psych
7	24	F	L	Knife	Upper arm (subtotal)	Deceased	NA – H/o mental illness	NA	NA	Rogers (1988)	Pathology
8	26	M	L	Electric saw	Hand	Y	Psychotic Depression	Y	Y	Scholzman (1998)	Psych
9	52	M	R	Circular saw	Hand	Y	Psychotic Depression	Y	Y	Scholzman (1998)	Psych
10	34	M	L	Knife	Hand (radiocarpal)	NR	Schizophreniform Disorder	Y	Y	Tavcar (1999)	Psych
11	29	M	L	Knife	Wrist	NR	Schizophrenia	Y	N	Kobayashi (2002)	Psych
12	51	M	L	Axe	Hand	NR	Body Integrity Identity Disorder	N	N	Sorene (2006)	Surgery
13	41	M	N/A	Power saw	Forearm	N	Bipolar	N	N	Schwartz (2009)	Psych
14	40	M	N/A	Power saw	Forearm	N	Depression	N	N	Schwartz (2009)	Psych
15	22	M	N/A	Dropsaw	Hand	NR	Depression	Y	Y	Crawford (2016)	Psych
16	20	M	N/A	Meat cleaver	Forearm	N	Bipolar I	Y	N	Van Bezooyen (2018)	Psych

One female, who died at the scene, was known to have a history of psychiatric hospitalization and a previous suicide attempt (Table 1) [12]. For the 15 patients that presented to the hospital, 13 patients (87%) were diagnosed with psychiatric disorders including depression (n = 6), bipolar (n = 2), and schizophrenia (n = 5). Of these 13 patients, 10 patients were additionally diagnosed with psychosis. The presence of a psychiatric diagnosis was not reported in one patient. Another patient presented with a diagnosis of body integrity identity disorder (BIID; Table 1), not currently classified as a mental disorder in the Diagnostic and Statistical Manual of Mental Disorders, Fifth Edition (DSM-5) [16]. BIID is an extremely rare psychological condition in which patients possess a longstanding, intense desire for limb amputation [16].

Ten patients (67%) underwent replantation and five patients (33%) did not. Eight of the replantation patients were diagnosed with psychosis. The presence (or absence) of psychosis was not reported in two of the replantation patients. Nine of the replants were performed for amputations at the hand/wrist level; one of the replants was done for a forearm level amputation. Postoperative data for the patients who underwent replantation is presented in Table 2. All of the replants survived. Inpatient psychiatry admission was documented as a component of the postoperative care in nine of the replant cases and not reported in one of the cases. Two of the 10 replantation patients were reported to be compliant with hand therapy, three of 10 patients were poorly compliant/noncompliant, and one patient was partially compliant. Hand therapy compliance was not reported in four of 10 patients.

**Table 2:** Replantation patients The patients with hand replantation’s post-operative data NR, not required; Inpt Psych, inpatient psychiatry [11,13-14,18]

Case #	Hand/Extremity Survival (Y/N)	Follow up	Postoperative Inpatient Psychiatry Admit	Hand Therapy Compliance	Patient Subjective	Functional Outcome
1	Y	1 year	NR	NR	NR	“extremely useful extremity”
2	Y	2 years	Inpt Psych	Poor	Satisfied	Picks up large objects, satisfactory grip
3	Y	NR	Inpt Psych	Poor	Satisfied	“useful function”
4	Y	NR	Inpt Psych	NR	NR	“continued to improve”
5	Y	NR	Inpt Psych	noncompliant	Pleased	“almost full function”
6	Y	NR	Inpt Psych	NR	NR	NR
8	Y	NR	Inpt Psych	Y	Pleased	“functioning well”
9	Y	NR	Inpt Psych	Partial	NR	“suboptimal”
10	Y	13 months	Inpt Psych	NR	NR	“favorable, moves fingers”
15	Y	NR	Inpt Psych	Y	NR	“good functional recovery”

Follow-up time was not consistently reported with only three of the replantation patients with follow-up beyond the immediate postoperative period. Four patients (40%) were pleased or satisfied with the replantation outcome. Patient subjective replantation outcome was not reported in the other six patients (60%). Detailed functional outcome data were not reported; only limited descriptions of function were available and were noted to be favorable in eight of 10 replantation patients. 

Of the 15 patients who presented to the hospital, five patients (33%) did not undergo replantation. In three patients, the amputated part was in poor condition for replantation. In two other patients, there was a prolonged time period to hospital presentation (>12 hours after forearm-level amputations). For the patients who did not undergo replantation, there was no mention of whether they were ultimately fitted with upper extremity prostheses.

Discussion

Complete hand/upper extremity amputations are severe injuries with potentially devastating functional sequela. Limb viability rates following upper extremity replantation have been reported to be as high as 80% to 94%; however, patients who undergo upper extremity replantation exhibit increased utilization of resources including longer length of hospital stay, higher rates of postoperative complications and greater rates of secondary surgical revisions [2,20-22]. Although restoration of perfusion with the survival of the replanted extremity is paramount and has been used as a determinant of a positive outcome, the anticipated functional result of the replanted limb deserves consideration and should exceed that expected from revision amputation and prosthetic fitting to rationalize the associated resource expenditure [23-24]. The objective functional benefit of upper extremity replantation compared to revision amputation and prosthetic fitting has been reported in the literature [24]. Furthermore, studies have demonstrated superior patient-reported outcomes with regard to subjective function as well as overall satisfaction compared to patients who underwent amputation and prosthetic rehabilitation [3,23].

The question then becomes whether these successful outcomes for upper extremity replantation can be anticipated in patients who present with deliberate, self-inflicted upper extremity amputations. Although a rare act, self-amputation of the hand/upper extremity could be underreported since patients may feel embarrassed by the mechanism of injury and aren't forthcoming about how the event occurred. Its presence in the literature has been limited to case reports and small series of patients. In our literature review, most of the cases were reported in the psychiatry literature (13/16, 81%) and focus predominantly on the acute mental health treatment of these patients with minimal emphasis on operative details and important postoperative follow-up data such as hand therapy compliance issues, subjective patient outcomes, and functional outcomes.

Self-inflicted amputation has often been considered a relative contraindication to replantation because of concerns related to future self-harm, in addition to other postoperative compliance issues, such as unreliable participation in rehabilitation efforts and poor physician follow-up [2]. The presence of a major psychiatric illness or mental instability has also been regarded as contraindications to replantation by some surgeons [5-6,25]. The responsible expenditure of healthcare resources must be taken into account as well, similar to all cases where replantation is considered. Based on our literature review of individuals with intentional self-amputation of the upper extremity, the decision to proceed with the replantation of an intentional upper-extremity amputation can be challenging. Appropriate patient selection is important, and emergent psychiatric consultation at the time of surgical evaluation may help guide decision-making. If replantation is performed, multidisciplinary care is essential and consists of postoperative surgical care and monitoring, ongoing psychiatric stabilization and treatment, and early hand therapy for rehabilitation of the replanted limb. Early institution of hand therapy with patient education and participation is of paramount importance since patients are often transferred to outside psychiatric facilities where an experienced hand therapist may not be available.

Our reported case demonstrates that it is possible to obtain excellent functional results with high patient satisfaction following upper extremity replantation as the result of a self-inflicted injury in a patient with mental illness. Nevertheless, based on the lack of available data in the literature, definitive recommendations concerning upper limb replantation in this population cannot be made. More detailed studies are required in this unique group of patients to evaluate long-term functional and patient-reported outcome data. Any decision to replant in this patient population likely needs to be carefully weighed considering several factors: the current psychiatric status of the patient, the anticipated commitment of the patient to several months of intensive hand therapy and to addressing ongoing psychiatric issues (including medication compliance and refrain from further self-harm of the replanted part). Also, the patient should be informed that future secondary surgery is likely following upper extremity replantation [26-27].

Limitations of our literature review include incomplete capture of additional cases of self-amputation that may be present within larger series of upper extremity amputations where detailed data and surgical outcomes were not accessible for this subset of patients. For example, in a series of 62 patients with traumatic upper extremity amputations reported by Larson et al., there were five patients (within a replantation group of 20) that underwent attempted replantation for self-inflicted injuries [2]. No significant statistical difference was noted in replantation success in self-inflicted amputations versus the non-self-inflicted group. We did not include these patients in our review. Another significant drawback to the literature review is that we were unable to obtain consistent objective functional data or patient-reported outcome data for patients that underwent replantation. Also, improved reporting of hand therapy compliance data would have been useful to better understand the level of patient commitment following such a major operative procedure in this patient population.

## Conclusions

Self-amputation of the hand and upper extremity is an extreme and uncommon act. Patients often present with complicated psychiatric issues or even frank psychosis. A review of the literature revealed that these patients often undergo replantation despite these associated psychiatric issues and our case shows that excellent outcomes are possible in these patients. A multi-disciplinary approach with the inpatient psychiatry team is vital for early functional rehabilitation to occur, and to allow appropriate prognostic estimates in these unique patients. Further evaluation of this patient population would be beneficial to better assess patient-reported outcomes and the functional benefits of replantation and allow more definitive recommendations on how to manage and optimize outcomes.
